# Enhanced efficacy and specificity of epithelial ovarian carcinogenesis by embedding a DMBA-coated cloth strip in the ovary of rat

**DOI:** 10.1186/1757-2215-5-21

**Published:** 2012-09-03

**Authors:** Yiping Huang, Wei Jiang, Yisheng Wang, Yufang Zheng, Qing Cong, Congjian Xu

**Affiliations:** 1 Obstetrics and Gynecology Hospital, Department of Obstetrics and Gynecology of Shanghai Medical School, and Institute of Biomedical Sciences, Fudan University, Shanghai, People’s Republic of China; 2 Shanghai Key Laboratory of Female Reproductive Endocrine Related Diseases, Shanghai, People’s Republic of China; 3 School of Life Sciences, Fudan University, Shanghai, 200433, People’s Republic of China; 4 Department of Gynecology, Obstetrics and Gynecology Hospital, Fudan University, 419 Fangxie Road, Shanghai, 200011, People’s Republic of China

**Keywords:** Ovarian cancer, Carcinogenesis, DMBA, Animal model, Rat

## Abstract

**Background:**

Ovarian cancer is predominant of epithelial cell origin and often present at an advanced stage with poor prognosis. Most animal models of ovarian carcinoma yield thecal/granulose cell tumors, rather than adenocarcinomas. The best reported induction rate of adenocarcinoma in rats is 10-45% by an ovarian implantation of 7, 12-dimethylbenz[a]anthracene (DMBA) coated silk suture. We provided an improved procedure to construct the model by the ovarian implantation of DMBA-coated cloth strip.

**Methods:**

A sterile suture (as S group) or a piece of cloth strip (as CS group) was soaked in DMBA before ovarian implantation in Wistar rats. Tumor size, incidence rate and pathological type were analyzed.

**Results:**

Ovarian tumors in rats of CS group were first noted at 16 wk post implantation and reached a cumulative incidence of 75% (96/128) at 32 wk, while the tumor incidence rate in S group at 32 wk was only 46.25% (37/80). The tumor size in CS group (3.63 ± 0.89 cm) was larger than that of S group (2.44 ± 1.89 cm) (*P* < 0.05). In CS group, there were only two types of tumor formed: adenocarcinoma (90/96) and sarcoma (6/96). While in S group, there were different types, including adenocarcinoma (21/37), squamous carcinoma (3/37), granulosa cell tumor (3/37), sarcoma (4/37), undifferentiated carcinoma with no adeno character (2/37), benign ovarian tumor (2/37), and malignant teratoma (1/37).

**Conclusion:**

The model in our study yields much higher incidence and specificity of epithelial derived tumors and showed histological similarities to human ovarian cancers, which would be more suitable for therapeutic research.

## Backgrounds

Epithelial ovarian cancer is a leading cause of female cancer mortality in the world [1]. In contrast to other women-specific cancers, like breast and uterine carcinomas, where death rates have fallen in recent years, ovarian cancer cure rates have remained relatively unchanged over the past two decades [2]. Ovarian adenocarcinomas account for 85-90% of all cancers of the ovary [3]. Effective detection and treatment of ovarian cancer remains a significant clinical challenge. The exactly initiating cell population for epithelial ovarian carcinoma (EOC) remains to be defined. Different evidences have suggested that EOC originate from the ovarian surface epithelium, inclusion cysts lined [4-7] or alternatively, the fallopian tube epithelium [8] or components of the secondary Müllerian system, including the epithelial cells of the rete ovarii, paraovarian/ paratubal cysts, endosalpingiosis, endometriosis or endomucinosis [9-13].

Because the results of ovarian carcinoma treatment are still far from optimal, animal models are still needed to study the human EOC. Even though spontaneous ovarian tumors in rodents have been reported [14], the paucity of these cases precludes their use in modeling ovarian cancer. Therefore, much effort has been put into developing relevant animal models for ovarian cancers. One such model involves the use of the carcinogen, 7, 12-dimethylbenz[a]anthracene (DMBA), a polycyclic aromatic hydrocarbon that induces carcinogenic mutations by forming DNA adducts [15]. Incidence of ovarian adenocarcinoma induction by DMBA varies between 10 to 45% [16,17], which probably due to the strain difference of rat employed, the chemical form of the DMBA utilized and the route of drug administrated in those studies.

Direct implantation of carcinogen appears to be critical as a single intragastric instillation or intravenous injection of DMBA in mice yields a comparable total tumor incidence, even though virtually most of the tumor exhibited a stromal tumor histology [18]. The traditional drug-containing silk suture implants would inevitably damage the stroma of ovary, leading to the diverse of pathological types of ovarian tumors in addition to epithelial tumors, such as sarcoma, granular cell tumor, etc. We report here that 75% tumor incidence at the end of 32 wk and 93.75% (90/96) of all tumors diagnosed as adenocarcinoma by implanting a piece of cloth strip soaked with high purity DMBA onto the surface of Wistar rat ovary. To our knowledge, this is the first report to use a cloth strip embedding to the surface of ovary to induce ovarian cancer.

## Material and methods

### Tumor induction procedures and evaluation

**Figure 1:**
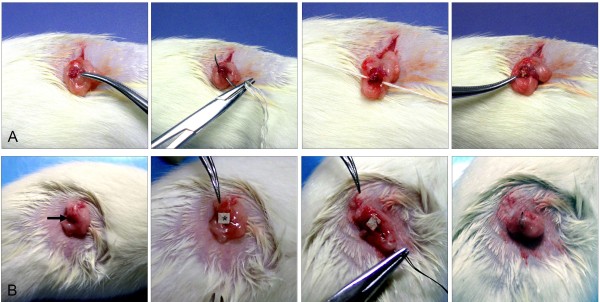
**Operation procedure for animals.A**: S group. **B**: CS group. DMBA was heated to 124°C. A central portion of 3–0 silk suture or a piece of cloth strip was immersed in the melted DMBA; sutures contained approximately 1 mg per centimeter of the carcinogen on average and each cloth strip contained approximately 1.5 mg of the carcinogen on average, as calibrated by a micro-chemical balance. Rats were anesthetized by intraperitoneal injection of chloral hydrate at 50 mg/kg. Then both ovaries were surgically exposed, and a DMBA-coated suture (S group, 80 rats) (**A**) or a DMBA-coated cloth strip (CS group, 128 rats) (**B**) was then inserted or packed into both ovaries and closed with the surrounding fatty substance. The arrow referred to the ovary of rat.

Five-week-old female Wistar rats (purchased from the Experimental Animal Center, Chinese Academy of Science, SYXK (shanghai) 2004–0011) were housed five per cage and acclimated to the animal room for one wk before surgery and received food and tap water *ad libitum*. The room was controlled for a constant temperature (22 ± 2°C) and relative humidity (50 ± 20%) with a 12 h light/dark cycle. 7,12-dimethylbenz(a)anthracene (DMBA) (Sigma Chemical Co, St Louis, MO) was heated to 124°C, which is the fusion point of the carcinogen. A central portion of 3–0 silk suture or a piece of cloth strip (0.5 cm × 0.5 cm) was immersed in the melted DMBA. The sutures contained approximately 1 mg per centimeter of the carcinogen on average and the cloth strip contained approximately 1.5 mg of the carcinogen on average, as calibrated by a micro-chemical balance. Rats were anesthetized by intraperitoneal injection of chloral hydrate (Shanghai No.1 Biochemical & Pharmaceutical Co, Shanghai, China) at 50 mg/kg. Then both ovaries were surgically exposed, and a DMBA-coated cloth strip (CS group, 128 rats) or a DMBA-coated suture, as a control group (S group, 80 rats) was then packed or inserted into both ovaries and closed with the surround fatty substance (Figure 1A and 1B). An antibiotic (10^5^ units of benzylpenicillin potassium) was administered intraperitoneally for prophylaxis against infection before the abdominal wall was closed. Tumor number, size and volume were determined weekly after the first operation in both groups by palpating the abdominal wall of rat.

This study was performed in strict accordance with the recommendations in the Guide for the Care and Use of Laboratory Animals of the National Science and Technology Committee. All protocols were approved by the Biomedical Research Ethics Committee of Shanghai Institute for Biological Science of Chinese Academy of Sciences. Every effort was made to minimize suffering.

### Preparation and analysis of tissues and histology

All rats were euthanized by CO_2_ inhalation followed by cervical dislocation, necropsied and examined for gross abnormalities. Pathologically altered organs, entire reproductive tracts and representative specimens of multiple organs and tissues, including the brain, lung, liver, kidney, spleen, pancreas and intestine were removed at necropsy, fixed in 10% (v/v) neutral buffered formalin overnight, transferred to 70% ethanol and paraffin-embedded. In rats with evident tumor, specimens of the tumor tissue were also excised, snap frozen in liquid N2 and stored at −80°C. For histological analysis, 5 μm formalin fixed paraffin embedded tissue sections were cut for hematoxylin and eosin stain (H&E staining). Histopathological analysis was performed by a pathologist with expertise in human and murine malignancies. The tumors were subtyped according to the histologic characteristics of neoplastic cells (Table 1) and the results of immunostain.

**Table 1:** Histologic subtyping of DMBA-induced ovarian carcinomas in rats

**Tumor subtypes**		**Histologic features**
Adenocarcinoma	Serous carcinoma	An adenocarcinoma characterized by a pattern of papillae with cellular budding
	Endometrioid carcinoma	An adenocarcinoma composed of simple or pseudostratified epithelial cells, or occasionally with squamous differentiation
	clear cell carcinoma	An adenocarcinoma mainly composed of hobnail cells
	mucinous carcinoma	An adenocarcinoma composed of endocervical-like and intestinal type cells
Granulosa cell tumor		A neoplasm composed of granulosa cells
Squamous carcinoma		A carcinoma composed of squamous epithelial cells
Sarcoma		A neoplasm composed of malignant nonepithelial cells
Malignant teratoma		Teratoma with any malignant contents
Undifferentiated carcinoma		A carcinoma with no Specifically differentiated cells
Benign ovarian tumor		All tumors without malignant characters

### Immunohistochemistry

Paraffin-embedded sections for immunohistochemistry were dewaxed, rehydrated and processed for antigen unmasking by heating to near boiling in citrate buffer (0.01 M, pH 6.0, 30 min) in a microwave oven, then allowed to cool for 20 min. Following this and all subsequent incubations except for the blocking serum, the slides were washed in phosphate-buffered saline (PBS, 3 × 5 min). Endogenous peroxidase activity was blocked by hydrogen peroxide incubation (0.3% in methanol) for 5 min. After incubation for 30 min in blocking serum (diluted 1:50 in PBS) appropriate to each secondary antibody, the slides were incubated with primary antibody at 4°C overnight (Primary antibodies were omitted as negative controls.) The primary antibodies used were as follows: polyclonal anti-rat Pan-CK (Santa Cruz Biotechnology, Santa Cruz, CA, USA), polyclonal anti-rat inhibin A (Santa Cruz Biotechnology, Santa Cruz, CA, USA),and polyclonal anti-rat vimentin (Abcam, ). Detection of primary antibody proceeded with the binding of biotinylated secondary antibody (diluted 1:200 for 10 min at room temperature) followed by avidin-biotin peroxidase complex (Vectastain Elite, Vector Laboratories, Burlingame, CA) and diaminobenzidine (DAB) chromagen substrate. Dark brown structures indicated positive immunostaining.

### Statistical analysis

The comparison of distribution of variables between two groups was made using the *t*-test (SPSS Statistics v13.0, Chicago, IL), with significance set at a *P* value of less than 0.05.

## Results

### Incidence of ovarian cancers

**Figure 2:**
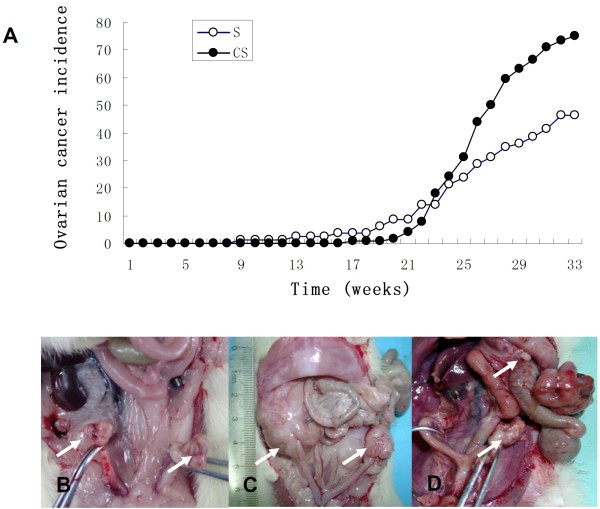
**The incidence of ovarian cancer of DMBA-treated rats and representative images of the tumors formed.** Tumors were detected by palpating the abdominal wall of rats once a week for a total of 32 weeks to monitor tumor progression and validated by a histological examination. Open circles, rats of the S group; closed circles, rats of the CS group (**A**). Tumors were usually a single invasive nodule (**B**, **C**), some were intraperitoneal spread or seeding of tumor (**D**), with or without ascites. Arrows refers to the tumor.

Thirty-two wk post DMBA treatment, rats in CS group had developed significantly more advanced tumors (larger ovarian tumors) compared with that of S group. On an average of 32 wk post DMBA treatment, 75% of the rats in CS group (96/128) had developed tumors; whereas only 46.25% (37/80) of the rats in the S group had developed tumors (*P* < 0.001). Tumors in both groups were mainly solid in structure, although a few tumors had a cystic cavity containing an abscess; some were intraperitoneal spread or seeding of tumor, with or without ascites. As shown in Figure 2.

### Morphologic features of ovaries after DMBA treatment

**Figure 3:**
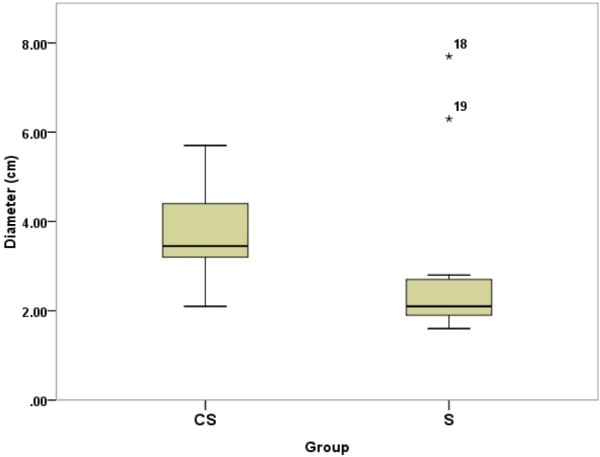
**Boxplots showing the tumor size between two groups.** The tumor size in CS group (3.63 ± 0.89 cm) was larger than that of S group (2.44 ± 1.89 cm) (*P* < 0.05), which was measured by counting the greatest dimension of the tumors. The number 18 and 19 in the figure indicate the outlier value of tumor size in the S group, referred to size 7.3 and 6.3 cm, respectively.

Morphologically, rats with tumors had an enlarged abdomen. The tumors presented as large masses with reddish color from the ovaries on both sides, and aggressively invaded surrounding organs including the spleen, intestine, kidney, and uterus, including some with bloody ascites. The mean greatest dimension of the tumors in CS group (3.63 ± 0.89 cm) was larger than that of S group (2.44 ± 1.89 cm) (*P < 0.05) (Figure 3).

### Histological features of induced tumor

**Figure 4:**
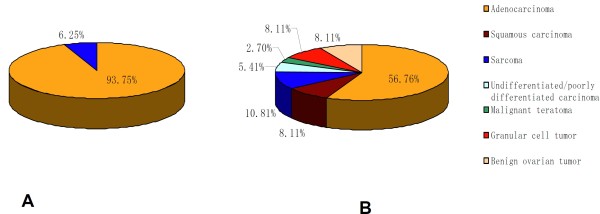
**Histological distribution of DMBA-induced carcinoma in two groups of rats.** In CS group (**A**), tumor histology included two types only: adenocarcinoma (90/96) and sarcoma (6/96). While in S group (**B**), tumor histology was distributed as adenocarcinoma (21/37), squamous carcinoma (3/37), granulosa cell tumor (3/37), sarcoma (4/37), undifferentiated carcinoma with no adeno character (2/37), benign ovarian tumor (2/37), and one malignant teratoma.

**Figure 5:**
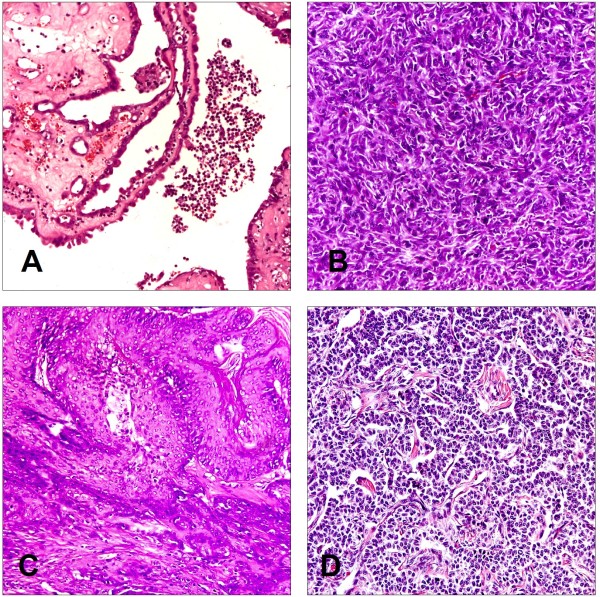
**Histopathology of ovarian tumors in two groups of rats.** Formalin-fixed tumors were sectioned and stained to determine cellular origin of the respective tumor and analysed in a blinded fashion by a pathologist. Representative tumor samples are shown as adenocarcinoma (**A**), sarcoma (**B**), squamous carcinoma (**C**), and granulosa cell tumor (**D**). Adenocarcinoma cells are arranged with glandular structures in back-to-back nests which are separated by Lumina. The nuclear/cytoplasmic ratio is high in these cells. The nucleus shows obvious pleomorphism, granular chromatin, and large plump nuclei with prominent nucleoli. Sarcoma cells are arranged in bundles and streams and are in an overall spindle shape. Multifocal squamous cell differentiation with marked keratinization are observed in squamous carcinoma. The tumor cells were microscopically similar to granulosa cells of normal follicles and numerous mitosis suggested a rapid growth in granulosa cell tumor. (H & E stain, 200×).

The majority of tumors in both groups were adenocarcinoma. The adenocarcinoma could be categorized as a mixed carcinoma, in which the lining epithelial cells were composed of flattened or cuboidal cells resembling ovarian surface epithelium, hobnail cells, and columnar cells, often with pseudostratified nuclei. Histologically, 93.75% (90/96) of the ovarian tumors in CS group are adenocarcinomas; the remaining tumors (6/96) displayed typical features of ovarian sarcomas (Figure 4A). While in S group, the histological type of induced tumor included: adenocarcinoma (21/37), squamous carcinoma (3/37), granulosa cell tumor (3/37), sarcoma (4/37), undifferentiated carcinoma with no adeno character (2/37), benign ovarian tumor (2/37), and malignant teratoma (1/37) (Figure 4B). Representative and typical tumor samples are shown as adenocarcinoma, sarcoma, squamous carcinoma, and granulosa cell tumor in Figure 5.

### Immunohistochemistry of induced tumors

**Figure 6:**
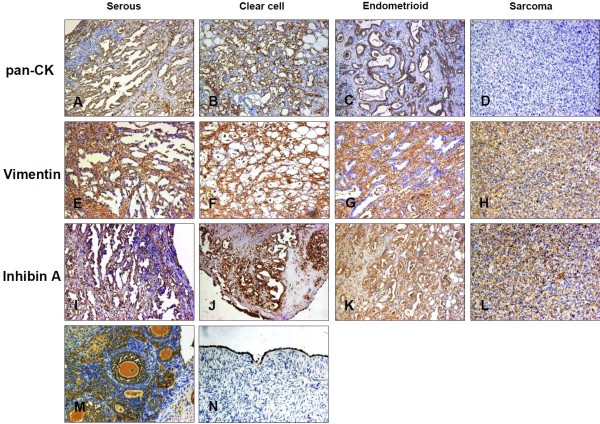
**Immunohistochemical stain of pan-CK, vimentin and inhibin A in rat ovarian tumor.** Pan-CK was expressed in (**A**) serous adenocarcinoma, (**B**) clear cell adenocarcinoma(**C**) endometroid adenocarcinoma, and (**N**) normal ovarian surface epithelium but not in (**D**) sarcoma. (200×). Vimentin was expressed in the stroma of (**E**) serous adenocarcinoma, (**F**) clear cell adenocarcinoma and (**G**) endometroid adenocarcinoma, but in (**H**) sarcoma cancer cell. (200×). Inhibin A was strongly expressed in (**I**) serous adenocarcinoma, (**J**) clear cell adenocarcinoma, (**K**) endometroid adenocarcinoma, and (**M**) granulosa cell of normal rat ovarian tissue, but weakly in (**L**) sarcoma. (200×).

To confirm histologic observations, we employed a number of immunohistochemical reagents for epithelial, grunulosa or mesenchymal cell types, such as pan-CK, vimentin and inhibin A (Figure 6). In adenocarcinoma, pan-CK staining of epithelial cells was most often diffuse but also appeared as localized to a luminal boundary. Epithelial cell staining of vimentin appeared as perinuclear, polar, or was absent from epithelial cells in more differentiated ductal structures and present only in the surrounding stroma. Inhibin A was strongly expressed in adenocarcinoma and weekly in sarcoma. There was no staining in the sarcoma cells for pan-CK while vimentin were positive.

## Discussion

In past, the DMBA treatment does not always successfully produce ovarian carcinoma with the rate of incidence at approximately 50% [19]. To date, a lot of reports available on overall DMBA-induced carcinoma in mice or rat have shown reproducible ovarian neoplasms with different delivery methods (gavage versus coated suture), dosage, length of exposure, and length of time before collection of tissues. In one study, C57BL6 mice received daily doses of DMBA by gavage for 3 wk; 71% of animals developed granulosa cell tumors after one year [20]. In another study, mice of a C57BL6; FVB/NCr background received a weekly dose of DMBA by gavage for 6 wk; 6 months after treatment, 27.3% (12/44) of mice developed ovarian neoplasms, of which 58% were granulosa cell tumors [21]. More recently, a study assessing the incidence of cancers in mice with a *P5*3 (*p53*^Ala135Val/wt^) mutation found that 80% of mice that received implants of DMBA-coated suture into their ovaries developed ovarian tumors, of which 50% were adenocarcinomas when evaluated 3 months after implantation [22].

Generally, traditional immunohistochemical markers used for the distinction of epithelial, stromal and granulosa ovarian tumor, such as CK, vimentin and inhibin A can be useful, and sometimes, they may show overlapping patterns of expression [23,24]. For example, vimentin could be immunostained in mucinous tumor, serous tumor, and low-grade endometrioid tumor [25-27]; inhibin A could be strongly expressed in epithelial ovarian cancer and other types of ovarian malignancies [25,26]. In our study, we determined the subtype of induced ovarian cancer by histologic observations in combination with immunohistochemistry. CK was expressed in the cancer cell of adenocarcinoma and normal ovarian surface epithelium but not in sarcoma and stromal. Although inhibin A was thought to be the specific marker of grunulosa cell, we detected it in the most of adenocarcinoma and some sarcoma cells in our induced tumors. This may due to some biological differences between the ovarian cancer of rat and human. Further investigations are required to address the discrepancies.

Compared with the ovaries of human beings, spontaneous development of epithelial neoplasms is rare in the ovaries of rodents [27,28]. One postulated reason for such low incidence is that the rat ovary is completely enveloped by a membranous pouch, which protects the surface epithelium against the effects of local carcinogens [29]. In our study, as shown in Figure 4, most of ovarian tumors induced in the embedded cloth strip group are adenocarcinoma and comfirmed by immunohistochemistry (Figure 6) while the silk suture group contained quite a lot of non-epithelial type tumors. The distinctly different outcome of two groups may due to the procedure during operation. In CS group, we maximally kept the inner part of ovaries uninjured by only dissecting their membranous pouch and let the surface cell exposed to drugs. While in S group, we inevitably injured the deep tissue of the ovaries when inserting the DMBA-coated suture by a needle to allow the drug penetrate into the tissues besides surface. Our results also provided further evidence that the origin of ovarian epithelial cancer derived from ovarian surface cell, and other types of cancer may originate from middle layer of ovary.

Intriguingly, the tumor size in our CS group had a lower standard deviation (S.D.) value than that of S group (0.89 vs. 1.89) (Figure 3). Thus, the cloth strip model generates a more homogeneous ovarian epithelial cancer model. It should provide a more suitable animal model for the research of ovarian cancer than any chemical-induced model reported previously.

## Conclusions

In summary, the results of this study indicate that the DMBA cloth strip model in rat yields high incidence and specificity of epithelial derived tumors histologically similar to human EOC and should be suitable for testing preventive or therapeutic agents for EOC.

## Abbreviations

DMBA: 7, 12-dimethylbenz[a]anthracene; CK: Cytokeratin; EOC: Epithelial ovarian carcinoma.

## Competing interests

The authors declare that they have no competing interests.

## Authors’ contributions

YH, WJ and CX participated in all aspects of the study, from design to laboratory performance, and manuscript writing. YW performed immunohistochemistry. YZ and QC participated in data analysis and editing of the manuscript. All authors have read and approved the manuscript.
